# 2-Trifluoro­methyl-1*H*-benzimidazol-3-ium tetra­fluoro­borate–2-trifluoro­methyl-1*H*-benzimidazole–water (1/1/1)

**DOI:** 10.1107/S1600536812010847

**Published:** 2012-03-17

**Authors:** Ming-Liang Liu

**Affiliations:** a Ordered Matter Science Research Center, Southeast University, Nanjing 211189, People’s Republic of China

## Abstract

The asymmetric unit of the title compound, C_8_H_6_F_3_N_2_^+^·BF_4_^−^·C_8_H_5_F_3_N_2_·H_2_O, consists of two 2-trifluoro­methyl­benzimidazole mol­ecules, each of which is protonated on a 50% basis, one tetra­fluoro­borate anion and a water mol­ecule. The two 2-trifluoromethylbenzimidazole mol­ecules thus exist as half-neutral half-cation entities. They are linked by N—H⋯N hydrogen bonds involving the half-occupancy hydrogens in each mol­ecule. The F atoms of one of the trifluoro­methyl groups are disordered over two sets of sites [in a 0.518 (14):0.482 (14) ratio], as are the F atoms of the tetra­fluoroborate anion [0.507 (14):0.493 (14) ratio]. The water mol­ecule is linked to one of the 2-trifluoro­methyl­benzimidazole mol­ecules *via* an N—H⋯O hydrogen bond.

## Related literature

The title compound was synthesized as part of our search for ferroelectric complexes. For background to ferroelectric complexes, see: Fu *et al.* (2011 ▶); Zhang *et al.* (2010 ▶). For related structures, see: Liu (2011*a* ▶,*b* ▶, 2012 ▶).
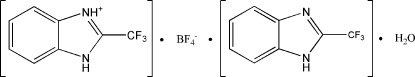


## Experimental

### 

#### Crystal data

C_8_H_6_F_3_N_2_^+^·BF_4_^−^·C_8_H_5_F_3_N_2_·H_2_O
*M* *_r_* = 478.11
Triclinic, 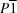

*a* = 8.7897 (18) Å
*b* = 10.947 (2) Å
*c* = 11.458 (2) Å
α = 92.48 (3)°
β = 96.59 (3)°
γ = 113.34 (3)°
*V* = 1000.8 (3) Å^3^
*Z* = 2
Mo *K*α radiation
μ = 0.17 mm^−1^
*T* = 293 K
0.36 × 0.32 × 0.28 mm

#### Data collection

Rigaku SCXmini diffractometer
Absorption correction: multi-scan (*CrystalClear*; Rigaku, 2005 ▶) *T*_min_ = 0.566, *T*_max_ = 0.640
10445 measured reflections
4581 independent reflections
2280 reflections with *I* > 2σ(*I*)
*R*_int_ = 0.039

#### Refinement

*R*[*F* ^2^ > 2σ(*F* ^2^)] = 0.066
*wR*(*F* ^2^) = 0.195
*S* = 1.03
4581 reflections
355 parameters
65 restraints
H-atom parameters constrained
Δρ_max_ = 0.30 e Å^−3^
Δρ_min_ = −0.21 e Å^−3^

### 

Data collection: *CrystalClear* (Rigaku, 2005 ▶); cell refinement: *CrystalClear*; data reduction: *CrystalClear*; program(s) used to solve structure: *SHELXS97* (Sheldrick, 2008 ▶); program(s) used to refine structure: *SHELXL97* (Sheldrick, 2008 ▶); molecular graphics: *PLATON* (Spek, 2009 ▶); software used to prepare material for publication: *SHELXTL* (Sheldrick, 2008 ▶).

## Supplementary Material

Crystal structure: contains datablock(s) I, global. DOI: 10.1107/S1600536812010847/go2047sup1.cif

Structure factors: contains datablock(s) I. DOI: 10.1107/S1600536812010847/go2047Isup2.hkl

Supplementary material file. DOI: 10.1107/S1600536812010847/go2047Isup3.cml

Additional supplementary materials:  crystallographic information; 3D view; checkCIF report

## Figures and Tables

**Table 1:** Hydrogen-bond geometry (Å, °)

*D*—H⋯*A*	*D*—H	H⋯*A*	*D*⋯*A*	*D*—H⋯*A*
N4—H4*A*⋯O1*W*	0.86	1.84	2.698 (3)	177
N1—H1⋯N3	0.86	1.86	2.718 (3)	175
N3—H3⋯N1	0.86	1.86	2.718 (3)	175

**Fig. 1.**
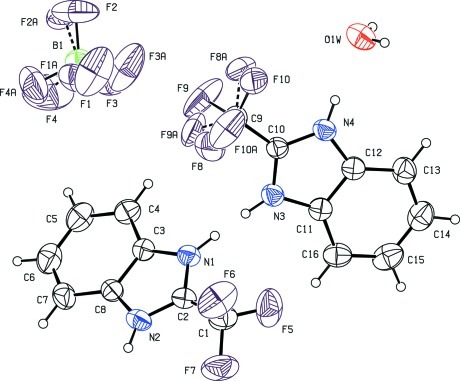
The molecular structure of the title compound, showing the atomic numbering scheme with 30% probability displacement ellipsoids. Both half hydrogens attached to N1 and N3 are included.

## supplementary crystallographic information

### Comment

Recently much attention has been devoted to crystals containing organic ions and inorganic ions due to the possibility of tuning their special structural features and their potential ferroelectrics properties (Fu *et al.*, 2011; Zhang *et al.*, 2010.). In our laboratory, the title compound has been synthesized to investigate to its potentialferroelectric properties. However, it was found that the dielectric constant of the compound as a function of temperature indicates that the permittivity is basically temperature-independent (*ε* = C/(T–T_0_)), suggesting that this compound is not ferroelectric or there may be no distinct phase transition occurring within the measured temperature (below the melting point).

The title compound, Figure 1, has an asymmetric unit which consists of two 2-trifluoromethylbenzimidazole molecules each of which is protonated on a 50% basis, one tetrafluoroborate anion,and a water molecule. The two trifluoromethylbenzimidazole moieties thus exist as a half neutral half cation entities. The two 2-trifluoromethylbenzimidazole molecules are hydrogen bonded together, on a 50/50 basis, by either the N1–H1···N3 or N3—H3···N1 hydrogen bonds, Table 1. One of the trifluoromethyl groups is disordered as is the tetraflouroborate anion. The water molecule is hydrogen bonded to one of the 2-trifluoromethylbenzimidazole molecules via the N4–H4A···O1W hydrogen bond Table 1. There are short N—H and O—H contacts to the F atoms of the anion but no analysis is made here because of the disorder in the anion.

### Experimental

0.144 g (1 mmol) of 2-trifluoromethyl-1*H*-benzimidazol was firstly dissolved in 30 ml of ethanol, to which 0.088 g (1 mmol) of fluoroboric acid was added to give a solution at the ambient temperature. Single crystals suitable for X-ray structure analysis were obtained after six days by the slow evaporation of the above solution in air.

### Refinement

H atoms were treated as riding atoms with N—H, 0.86Å, C—H(aromatic), 0.95 Å, with *U*_iso_ = 1.2Ueq(C) allowed to ride. The H atoms attached to the water molecule were refined as riding atoms at positions deteremined from a difference Fourier with *U*_iso_ = 1.5Ueq(O). An examination of a difference Fourier along the line of the N1 to N3 vector showed an elongated density peak. This was found to be best modelled as two half-hydrogen atoms attached to N1 and N3. All H atom positions were checked on a final difference Fourier.

The disordered trifluoromethyl group was modelled over two sites with restrained bonds and angles based on the average values found for the non-disordered trifluoromethyl group in the other molecule. The site occupancies were refined and restraints were applied to the thermal parameters. The terafluoroborate anion is also modelled as being disordered over two sites. The site occupancies were refined and restraints were applied to the bonds, angles and the thermal parameters.

### Crystal data

**Table:**

C_8_H_6_F_3_N_2_^+^·BF_4_^−^·C_8_H_5_F_3_N_2_·H_2_O	*V* = 1000.8 (3) Å^3^
*M**_r_* = 478.11	*Z* = 2
Triclinic, *P*1	*F*(000) = 480
Hall symbol: -P 1	*D*_x_ = 1.587 Mg m^−^^3^
*a* = 8.7897 (18) Å	Mo *K*α radiation, λ = 0.71073 Å
*b* = 10.947 (2) Å	θ = 0–25°
*c* = 11.458 (2) Å	µ = 0.17 mm^−^^1^
α = 92.48 (3)°	*T* = 293 K
β = 96.59 (3)°	Block, colourless
γ = 113.34 (3)°	0.36 × 0.32 × 0.28 mm

### Data collection

**Table:**

Rigaku SCXmini diffractometer	4581 independent reflections
Radiation source: fine-focus sealed tube	2280 reflections with *I* > 2σ(*I*)
Graphite monochromator	*R*_int_ = 0.039
CCD_Profile_fitting scans	θ_max_ = 27.5°, θ_min_ = 3.0°
Absorption correction: multi-scan (*CrystalClear*; Rigaku, 2005)	*h* = −11→11
*T*_min_ = 0.566, *T*_max_ = 0.640	*k* = −14→14
10445 measured reflections	*l* = −14→14

### Refinement

**Table:**

Refinement on *F*^2^	Secondary atom site location: difference Fourier map
Least-squares matrix: full	Hydrogen site location: inferred from neighbouring sites
*R*[*F*^2^ > 2σ(*F*^2^)] = 0.066	H-atom parameters constrained
*wR*(*F*^2^) = 0.195	*w* = 1/[σ^2^(*F*_o_^2^) + (0.073*P*)^2^ + 0.2894*P*] where *P* = (*F*_o_^2^ + 2*F*_c_^2^)/3
*S* = 1.03	(Δ/σ)_max_ < 0.001
4581 reflections	Δρ_max_ = 0.30 e Å^−^^3^
355 parameters	Δρ_min_ = −0.21 e Å^−^^3^
65 restraints	Extinction correction: *SHELXL97* (Sheldrick, 2008), Fc^*^=kFc[1+0.001xFc^2^λ^3^/sin(2θ)]^-1/4^
Primary atom site location: structure-invariant direct methods	Extinction coefficient: 0.020 (4)

### Special details

**Table:**

Geometry. All esds (except the esd in the dihedral angle between two l.s. planes) are estimated using the full covariance matrix. The cell esds are taken into account individually in the estimation of esds in distances, angles and torsion angles; correlations between esds in cell parameters are only used when they are defined by crystal symmetry. An approximate (isotropic) treatment of cell esds is used for estimating esds involving l.s. planes.
Refinement. Refinement of F^2^ against ALL reflections. The weighted R-factor wR and goodness of fit S are based on F^2^, conventional R-factors R are based on F, with F set to zero for negative F^2^. The threshold expression of F^2^ > 2sigma(F^2^) is used only for calculating R-factors(gt) etc. and is not relevant to the choice of reflections for refinement. R-factors based on F^2^ are statistically about twice as large as those based on F, and R- factors based on ALL data will be even larger.

### Fractional atomic coordinates and isotropic or equivalent isotropic displacement parameters (Å^2^)

**Table:**

	*x*	*y*	*z*	*U*_iso_*/*U*_eq_	Occ. (<1)
F5	−0.1135 (3)	0.6112 (3)	0.9237 (2)	0.1358 (10)	
F6	0.0425 (3)	0.6539 (3)	1.0868 (3)	0.1405 (11)	
F7	−0.1136 (3)	0.7537 (3)	1.0522 (3)	0.1382 (10)	
N1	0.1904 (3)	0.7918 (2)	0.8667 (2)	0.0594 (6)	
H1	0.1676	0.7165	0.8274	0.071*	0.50
N2	0.1829 (3)	0.9413 (3)	0.9962 (2)	0.0671 (7)	
H2A	0.1531	0.9753	1.0539	0.081*	
C1	−0.0170 (5)	0.7072 (4)	1.0035 (3)	0.0815 (10)	
C2	0.1171 (4)	0.8130 (3)	0.9543 (2)	0.0593 (7)	
C3	0.3121 (3)	0.9151 (3)	0.8501 (2)	0.0562 (7)	
C4	0.4274 (4)	0.9514 (3)	0.7713 (3)	0.0740 (9)	
H4	0.4338	0.8885	0.7171	0.089*	
C5	0.5317 (5)	1.0842 (4)	0.7770 (3)	0.0882 (11)	
H5	0.6102	1.1119	0.7251	0.106*	
C6	0.5231 (5)	1.1781 (4)	0.8579 (4)	0.0926 (12)	
H6	0.5951	1.2674	0.8580	0.111*	
C7	0.4121 (4)	1.1439 (3)	0.9378 (4)	0.0810 (10)	
H7	0.4078	1.2068	0.9931	0.097*	
C8	0.3070 (4)	1.0102 (3)	0.9311 (2)	0.0587 (7)	
N3	0.1071 (3)	0.5575 (2)	0.7327 (2)	0.0622 (6)	
H3	0.1364	0.6341	0.7717	0.075*	0.50
N4	0.1163 (3)	0.3728 (2)	0.6582 (2)	0.0627 (6)	
H4A	0.1544	0.3135	0.6429	0.075*	
C9	0.3685 (3)	0.5281 (2)	0.7895 (2)	0.0757 (9)	
C10	0.1977 (3)	0.4870 (3)	0.7266 (2)	0.0569 (7)	
F8	0.3784 (12)	0.5766 (11)	0.8977 (4)	0.130 (4)	0.518 (14)
F9	0.4883 (7)	0.6155 (10)	0.7417 (9)	0.163 (5)	0.518 (14)
F10	0.4064 (9)	0.4244 (5)	0.8009 (8)	0.105 (3)	0.518 (14)
F8A	0.4595 (8)	0.4758 (11)	0.7415 (10)	0.161 (5)	0.482 (14)
F9A	0.4470 (9)	0.6579 (3)	0.7890 (10)	0.117 (3)	0.482 (14)
F10A	0.3750 (11)	0.5061 (12)	0.9007 (4)	0.135 (4)	0.482 (14)
C11	−0.0450 (4)	0.4836 (3)	0.6635 (2)	0.0593 (7)	
C12	−0.0405 (4)	0.3665 (3)	0.6161 (2)	0.0602 (7)	
C13	−0.1752 (4)	0.2705 (3)	0.5431 (3)	0.0771 (9)	
H13	−0.1705	0.1930	0.5106	0.093*	
C14	−0.3154 (5)	0.2961 (4)	0.5213 (3)	0.0897 (11)	
H14	−0.4098	0.2332	0.4743	0.108*	
C15	−0.3204 (5)	0.4133 (5)	0.5675 (3)	0.0921 (11)	
H15	−0.4180	0.4270	0.5492	0.110*	
C16	−0.1879 (5)	0.5100 (4)	0.6388 (3)	0.0786 (9)	
H16	−0.1926	0.5884	0.6690	0.094*	
F1	0.8155 (16)	0.8437 (14)	0.8026 (9)	0.191 (5)	0.507 (14)
F2	0.9801 (12)	0.8884 (13)	0.6676 (9)	0.164 (5)	0.507 (14)
F3	0.7345 (11)	0.8971 (12)	0.6305 (8)	0.131 (3)	0.507 (14)
F4	0.9301 (13)	1.0476 (8)	0.7603 (10)	0.175 (4)	0.507 (14)
F1A	0.8505 (12)	0.8938 (8)	0.8232 (5)	0.105 (3)	0.493 (14)
F2A	1.0041 (11)	0.9229 (11)	0.6739 (11)	0.123 (3)	0.493 (14)
F3A	0.7326 (12)	0.8392 (17)	0.6363 (9)	0.208 (6)	0.493 (14)
F4A	0.875 (2)	1.0469 (10)	0.7068 (16)	0.233 (6)	0.493 (14)
B1	0.8652 (5)	0.9202 (4)	0.7116 (3)	0.0835 (11)	
O1W	0.2248 (3)	0.1817 (2)	0.6036 (2)	0.0972 (8)	
H1A	0.2132	0.1781	0.5299	0.146*	
H1B	0.1572	0.1131	0.6279	0.146*	

### Atomic displacement parameters (Å^2^)

**Table:**

	*U*^11^	*U*^22^	*U*^33^	*U*^12^	*U*^13^	*U*^23^
F5	0.121 (2)	0.1109 (18)	0.1217 (19)	−0.0148 (15)	0.0434 (16)	0.0023 (15)
F6	0.122 (2)	0.165 (2)	0.150 (2)	0.0578 (18)	0.0486 (17)	0.103 (2)
F7	0.125 (2)	0.139 (2)	0.180 (3)	0.0620 (17)	0.098 (2)	0.0385 (19)
N1	0.0647 (15)	0.0599 (15)	0.0577 (14)	0.0281 (12)	0.0147 (11)	0.0044 (11)
N2	0.0804 (17)	0.0739 (17)	0.0576 (14)	0.0435 (15)	0.0091 (13)	−0.0042 (13)
C1	0.081 (2)	0.090 (3)	0.085 (2)	0.039 (2)	0.032 (2)	0.021 (2)
C2	0.0667 (18)	0.0633 (19)	0.0581 (17)	0.0347 (15)	0.0152 (14)	0.0105 (14)
C3	0.0581 (17)	0.0588 (17)	0.0529 (16)	0.0254 (14)	0.0055 (13)	0.0079 (13)
C4	0.074 (2)	0.083 (2)	0.0670 (19)	0.0312 (19)	0.0200 (16)	0.0147 (17)
C5	0.076 (2)	0.093 (3)	0.093 (3)	0.026 (2)	0.0198 (19)	0.035 (2)
C6	0.077 (2)	0.067 (2)	0.126 (3)	0.0241 (19)	−0.003 (2)	0.028 (2)
C7	0.078 (2)	0.059 (2)	0.103 (3)	0.0329 (18)	−0.015 (2)	−0.0030 (18)
C8	0.0611 (17)	0.0600 (18)	0.0571 (16)	0.0299 (15)	−0.0016 (14)	0.0015 (14)
N3	0.0662 (15)	0.0575 (14)	0.0627 (14)	0.0228 (13)	0.0174 (12)	0.0043 (11)
N4	0.0675 (16)	0.0614 (15)	0.0619 (14)	0.0271 (12)	0.0180 (12)	0.0018 (12)
C9	0.066 (2)	0.071 (2)	0.084 (2)	0.0213 (18)	0.0118 (18)	−0.0024 (19)
C10	0.0589 (17)	0.0561 (17)	0.0551 (16)	0.0210 (14)	0.0150 (13)	0.0039 (13)
F8	0.117 (5)	0.162 (7)	0.106 (5)	0.075 (6)	−0.042 (4)	−0.067 (4)
F9	0.061 (3)	0.191 (8)	0.237 (10)	0.033 (5)	0.047 (5)	0.130 (8)
F10	0.078 (4)	0.092 (3)	0.151 (6)	0.050 (3)	−0.013 (3)	−0.003 (3)
F8A	0.065 (4)	0.194 (10)	0.207 (10)	0.049 (6)	0.009 (5)	−0.090 (8)
F9A	0.053 (4)	0.098 (4)	0.168 (7)	0.000 (3)	0.001 (3)	0.018 (4)
F10A	0.088 (4)	0.198 (9)	0.100 (5)	0.033 (6)	0.008 (4)	0.068 (5)
C11	0.0651 (18)	0.0645 (18)	0.0503 (15)	0.0260 (15)	0.0143 (14)	0.0116 (13)
C12	0.0653 (19)	0.0672 (19)	0.0472 (15)	0.0237 (15)	0.0153 (14)	0.0080 (13)
C13	0.080 (2)	0.082 (2)	0.0578 (18)	0.0223 (18)	0.0085 (16)	−0.0049 (16)
C14	0.078 (2)	0.114 (3)	0.062 (2)	0.025 (2)	0.0044 (17)	0.002 (2)
C15	0.081 (3)	0.135 (4)	0.070 (2)	0.054 (3)	0.0062 (19)	0.017 (2)
C16	0.085 (2)	0.091 (2)	0.072 (2)	0.048 (2)	0.0146 (19)	0.0147 (18)
F1	0.141 (6)	0.243 (10)	0.136 (7)	0.019 (8)	0.012 (5)	0.086 (8)
F2	0.181 (8)	0.289 (13)	0.104 (6)	0.188 (10)	0.009 (5)	−0.012 (6)
F3	0.102 (5)	0.214 (8)	0.092 (4)	0.093 (5)	−0.016 (4)	−0.020 (4)
F4	0.173 (7)	0.131 (6)	0.174 (8)	0.018 (5)	0.037 (6)	−0.071 (5)
F1A	0.134 (6)	0.129 (5)	0.060 (3)	0.063 (5)	0.018 (3)	−0.015 (3)
F2A	0.091 (4)	0.140 (6)	0.143 (8)	0.039 (4)	0.053 (5)	0.031 (5)
F3A	0.106 (5)	0.339 (14)	0.083 (4)	−0.008 (7)	0.013 (4)	−0.018 (7)
F4A	0.264 (14)	0.170 (7)	0.361 (18)	0.151 (8)	0.140 (12)	0.133 (9)
B1	0.087 (3)	0.095 (3)	0.076 (3)	0.043 (3)	0.015 (2)	0.010 (2)
O1W	0.117 (2)	0.0938 (18)	0.0889 (17)	0.0516 (15)	0.0183 (15)	−0.0090 (13)

### Geometric parameters (Å, º)

**Table:**

F5—C1	1.301 (4)	C9—F8A	1.306 (2)
F6—C1	1.305 (4)	C9—F10A	1.306 (2)
F7—C1	1.311 (4)	C9—F8	1.307 (2)
N1—C2	1.312 (3)	C9—F10	1.311 (2)
N1—C3	1.388 (4)	C9—F9A	1.312 (2)
N1—H1	0.8600	C9—C10	1.471 (3)
N2—C2	1.331 (3)	C11—C12	1.388 (4)
N2—C8	1.378 (4)	C11—C16	1.398 (4)
N2—H2A	0.8600	C12—C13	1.384 (4)
C1—C2	1.475 (5)	C13—C14	1.367 (5)
C3—C8	1.382 (4)	C13—H13	0.9300
C3—C4	1.387 (4)	C14—C15	1.386 (5)
C4—C5	1.371 (5)	C14—H14	0.9300
C4—H4	0.9300	C15—C16	1.369 (5)
C5—C6	1.385 (5)	C15—H15	0.9300
C5—H5	0.9300	C16—H16	0.9300
C6—C7	1.370 (5)	F1—B1	1.368 (7)
C6—H6	0.9300	F2—B1	1.332 (6)
C7—C8	1.379 (4)	F3—B1	1.324 (7)
C7—H7	0.9300	F4—B1	1.346 (7)
N3—C10	1.316 (3)	F1A—B1	1.332 (7)
N3—C11	1.383 (4)	F2A—B1	1.332 (7)
N3—H3	0.8600	F3A—B1	1.332 (7)
N4—C10	1.329 (3)	F4A—B1	1.361 (7)
N4—C12	1.380 (4)	O1W—H1A	0.8362
N4—H4A	0.8600	O1W—H1B	0.8349
C9—F9	1.305 (2)		
			
C2—N1—C3	106.4 (2)	F8A—C9—F9A	106.1 (3)
C2—N1—H1	126.8	F10A—C9—F9A	105.4 (3)
C3—N1—H1	126.8	F9—C9—C10	116.2 (4)
C2—N2—C8	108.2 (2)	F8A—C9—C10	114.7 (4)
C2—N2—H2A	125.9	F10A—C9—C10	114.2 (5)
C8—N2—H2A	125.9	F8—C9—C10	110.4 (4)
F5—C1—F6	107.6 (3)	F10—C9—C10	110.7 (3)
F5—C1—F7	106.9 (3)	F9A—C9—C10	108.2 (4)
F6—C1—F7	105.5 (3)	N3—C10—N4	112.2 (2)
F5—C1—C2	112.5 (3)	N3—C10—C9	124.2 (2)
F6—C1—C2	112.1 (3)	N4—C10—C9	123.6 (2)
F7—C1—C2	111.8 (3)	N3—C11—C12	108.4 (3)
N1—C2—N2	111.6 (3)	N3—C11—C16	130.7 (3)
N1—C2—C1	124.3 (3)	C12—C11—C16	120.9 (3)
N2—C2—C1	124.0 (3)	N4—C12—C13	132.0 (3)
C8—C3—C4	120.4 (3)	N4—C12—C11	105.6 (2)
C8—C3—N1	108.3 (2)	C13—C12—C11	122.3 (3)
C4—C3—N1	131.3 (3)	C14—C13—C12	116.3 (3)
C5—C4—C3	117.0 (3)	C14—C13—H13	121.8
C5—C4—H4	121.5	C12—C13—H13	121.8
C3—C4—H4	121.5	C13—C14—C15	121.6 (3)
C4—C5—C6	121.7 (3)	C13—C14—H14	119.2
C4—C5—H5	119.2	C15—C14—H14	119.2
C6—C5—H5	119.2	C16—C15—C14	122.9 (4)
C7—C6—C5	122.2 (3)	C16—C15—H15	118.6
C7—C6—H6	118.9	C14—C15—H15	118.6
C5—C6—H6	118.9	C15—C16—C11	115.9 (3)
C6—C7—C8	115.8 (3)	C15—C16—H16	122.0
C6—C7—H7	122.1	C11—C16—H16	122.0
C8—C7—H7	122.1	F3—B1—F2	111.2 (6)
N2—C8—C7	131.5 (3)	F2A—B1—F3A	109.3 (8)
N2—C8—C3	105.5 (2)	F2A—B1—F1A	115.8 (7)
C7—C8—C3	123.0 (3)	F3A—B1—F1A	112.5 (7)
C10—N3—C11	106.1 (2)	F3—B1—F4	110.9 (6)
C10—N3—H3	126.9	F2—B1—F4	111.7 (7)
C11—N3—H3	126.9	F1A—B1—F4	82.7 (7)
C10—N4—C12	107.6 (2)	F2A—B1—F4A	105.0 (7)
C10—N4—H4A	126.2	F3A—B1—F4A	107.9 (8)
C12—N4—H4A	126.2	F1A—B1—F4A	105.7 (7)
F8A—C9—F10A	107.6 (4)	F3—B1—F1	109.8 (7)
F9—C9—F8	107.6 (4)	F2—B1—F1	107.6 (7)
F9—C9—F10	106.5 (3)	F4—B1—F1	105.4 (6)
F8—C9—F10	104.6 (3)	H1A—O1W—H1B	112.4
			
C3—N1—C2—N2	−0.3 (3)	C12—N4—C10—C9	178.8 (2)
C3—N1—C2—C1	−178.0 (3)	F9—C9—C10—N3	−79.8 (7)
C8—N2—C2—N1	0.8 (3)	F8A—C9—C10—N3	−155.5 (8)
C8—N2—C2—C1	178.5 (3)	F10A—C9—C10—N3	79.6 (7)
F5—C1—C2—N1	−33.5 (5)	F8—C9—C10—N3	43.1 (6)
F6—C1—C2—N1	87.9 (4)	F10—C9—C10—N3	158.5 (5)
F7—C1—C2—N1	−153.8 (3)	F9A—C9—C10—N3	−37.4 (6)
F5—C1—C2—N2	149.1 (3)	F9—C9—C10—N4	101.1 (7)
F6—C1—C2—N2	−89.5 (4)	F8A—C9—C10—N4	25.4 (8)
F7—C1—C2—N2	28.8 (5)	F10A—C9—C10—N4	−99.4 (7)
C2—N1—C3—C8	−0.3 (3)	F8—C9—C10—N4	−135.9 (6)
C2—N1—C3—C4	179.1 (3)	F10—C9—C10—N4	−20.6 (6)
C8—C3—C4—C5	−1.1 (4)	F9A—C9—C10—N4	143.6 (6)
N1—C3—C4—C5	179.6 (3)	C10—N3—C11—C12	−0.1 (3)
C3—C4—C5—C6	0.3 (5)	C10—N3—C11—C16	179.6 (3)
C4—C5—C6—C7	0.9 (6)	C10—N4—C12—C13	−179.4 (3)
C5—C6—C7—C8	−1.2 (5)	C10—N4—C12—C11	0.3 (3)
C2—N2—C8—C7	179.7 (3)	N3—C11—C12—N4	−0.1 (3)
C2—N2—C8—C3	−0.9 (3)	C16—C11—C12—N4	−179.8 (3)
C6—C7—C8—N2	179.8 (3)	N3—C11—C12—C13	179.6 (3)
C6—C7—C8—C3	0.4 (4)	C16—C11—C12—C13	−0.2 (4)
C4—C3—C8—N2	−178.8 (3)	N4—C12—C13—C14	178.5 (3)
N1—C3—C8—N2	0.7 (3)	C11—C12—C13—C14	−1.1 (4)
C4—C3—C8—C7	0.7 (4)	C12—C13—C14—C15	1.6 (5)
N1—C3—C8—C7	−179.8 (3)	C13—C14—C15—C16	−1.0 (6)
C11—N3—C10—N4	0.3 (3)	C14—C15—C16—C11	−0.3 (5)
C11—N3—C10—C9	−178.8 (2)	N3—C11—C16—C15	−178.9 (3)
C12—N4—C10—N3	−0.4 (3)	C12—C11—C16—C15	0.8 (4)

### Hydrogen-bond geometry (Å, º)

**Table:**

*D*—H···*A*	*D*—H	H···*A*	*D*···*A*	*D*—H···*A*
N4—H4*A*···O1*W*	0.86	1.84	2.698 (3)	177
N1—H1···N3	0.86	1.86	2.718 (3)	175
N3—H3···N1	0.86	1.86	2.718 (3)	175
